# Resonance assignments of cytochrome MtoD from the extracellular electron uptake pathway of *sideroxydans lithotrophicus* ES-1

**DOI:** 10.1007/s12104-024-10180-8

**Published:** 2024-06-07

**Authors:** Anaísa Coelho, José M. Silva, Francesca Cantini, Mario Piccioli, Ricardo O. Louro, Catarina M. Paquete

**Affiliations:** 1 Instituto de Tecnologia Química e Biológica António Xavier (ITQB-NOVA), Universidade Nova de Lisboa, Av. da República (EAN), Oeiras, 2780-157 Portugal; 2 Department of Chemistry, Magnetic Resonance Center (CERM), University of Florence, Via L. Sacconi 6, Sesto Fiorentino, 50019 Italy; 3 Consorzio Interuniversitario Risonanze Magnetiche MetalloProteine (CIRMMP), Via L. Sacconi 6, Sesto Fiorentino, 50019 Italy

**Keywords:** NMR resonance assignment, Cytochromes, MtoD, Extracellular electron transfer, Ring current shifts

## Abstract

The contribution of Fe(II)-oxidizing bacteria to iron cycling in freshwater, groundwater, and marine environments has been widely recognized in recent years. These organisms perform extracellular electron transfer (EET), which constitutes the foundations of bioelectrochemical systems for the production of biofuels and bioenergy. It was proposed that the Gram-negative bacterium *Sideroxydans lithotrophicus* ES-1 oxidizes soluble ferrous Fe(II) at the surface of the cell and performs EET through the Mto redox pathway. This pathway is composed by the periplasmic monoheme cytochrome MtoD that is proposed to bridge electron transfer between the cell exterior and the cytoplasm. This makes its functional and structural characterization, as well as evaluating the interaction process with its physiological partners, essential for understanding the mechanisms underlying EET. Here, we report the complete assignment of the heme proton and carbon signals together with a near-complete assignment of ^1^H, ^13^C and ^15^N backbone and side chain resonances for the reduced, diamagnetic form of the protein. These data pave the way to identify and structurally map the molecular interaction regions between the cytochrome MtoD and its physiological redox partners, to explore the EET processes of *S. lithotrophicus* ES-1.

**Supplementary Information:**

The online version contains supplementary material available at 10.1007/s12104-024-10180-8.

## Biological context

Fe(II)-oxidizing bacteria play an important role in numerous biogeochemical cycles, including that of iron (Kappler and Straub 2005), and are currently being explored for the development of biotechnological processes such as bioelectrosynthesis where microbial metabolism is driven directly by electricity (Gupta et al. 2020; Karthikeyan et al. 2019; Summers et al. 2013). In their natural environments, these organisms perform extracellular electron transfer (EET), taking up electrons from Fe(II) outside of the cell, and transferring them to the quinone pool in the inner membrane. These pathways connect extracellular redox reactions to intracellular metabolic activity and are made up of redox and structural proteins that work together to transfer electrons between extracellular substrates and the cytoplasmic membrane (Bird et al. 2014; Jain et al. 2022b; Liu et al. 2012; Valdés et al. 2008; Zhou et al. 2022).

Although several Fe(II) oxidizers have been identified, the understanding of the electron transfer mechanisms involved in growth and energy conservation in Fe(II)-oxidizing bacteria lags far behind. This is primarily due to difficulties encountered in culturing various of these microorganisms that achieve only poor cell yields, as well as the lack of genetic systems for their manipulation in many cases (Ilbert and Bonnefoy 2013).

*Sideroxydans lithotrophicus* ES-1 is a freshwater chemolithoautotrophic Gram-negative bacterium able to oxidize Fe(II) at the cell surface. It was proposed that this organism relies on different pathways to perform EET, including the Mto pathway (Jain et al. 2022b; Zhou et al. 2022). In this pathway, it is proposed that the monoheme cytochrome MtoD shuttles electrons across the periplasmic space, between the outer membrane-associated decaheme cytochrome-porin complex MtoAB and the inner membrane oxidoreductase ImoA, a tetraheme cytochrome that belongs to the NapC/NirT family (Jain et al. 2022a, b). MtoA and MtoB are homologs of MtrA and MtrB, respectively. These proteins belong to the MtrCAB complex from the Fe(III)-reducing Gram-negative bacterium *Shewanella oneidensis* MR-1, known to be involved in efficient electron transport across the outer membrane (Zhong and Shi 2018). ImoA has been proposed to function as the quinone oxidoreductase during Fe(II) oxidation based on its sequence similarity with the tetraheme cytochrome CymA from *S. oneidensis* MR-1 (Liu et al. 2012). The gene coding for MtoA, MtoB, MtoD and ImoA are all clustered together, indicating that their protein products likely work collectively to facilitate electron transfer across the cell envelope (He et al. 2017; Liu et al. 2012). It has been demonstrated that these proteins exhibit modularity, incorporating elements from other porin-cytochrome pathways, including those identified in *S. lithotrophicus* ES-1 (Jain et al. 2022b; Zhou et al. 2022). This suggests that this organism relies on multiple interconnected EET pathways to facilitate Fe(II) oxidation (Jain et al. 2022b).

MtoD is a 117 amino acids monoheme *c*-type cytochrome that belongs to the class I of cytochromes *c* predicted to be located in the periplasm. Residues 1–27, forming the signal peptide, are cleaved and the mature protein, containing a Strep-II affinity tag, is a 98 aminoacids, 11 kDa protein. The atomic structure of this protein in the oxidized state was determined by X-ray crystallography. It revealed the *c*-type heme cofactor axially coordinated by two histidine residues, which is an uncommon feature for this class of cytochromes (Beckwith et al. 2015). The unusual bis-His axial heme ligation gives MtoD a lower reduction potential (155 ± 10mV (Beckwith et al. 2015) than typically observed for class I cytochromes *c* ( ~ + 200 to + 250 mV). To validate the proposed pathway and verify that MtoD can receive electrons from MtoA and transfer them to ImoA, interaction studies must be performed. NMR spectroscopy is a powerful technique to explore transient complexes and weak protein–protein interactions (Fonseca et al. 2013; Piccioli and Turano 2015; Ubbink 2012), providing both dynamic and structural information in different oxidation states of redox proteins in solution at atomic resolution (Fonseca et al. 2013; Morgado and Salgueiro 2022). However, to achieve this, a full assignment of the NMR signals of MtoD is mandatory not only to study interactions between MtoD and physiological partner proteins, but also to identify the interacting regions at the surface of MtoD. In this work, the complete assignment of the heme proton and carbon signals and a near-complete assignment of ^1^H, ^15^N and ^13^C backbone and side chain resonances for MtoD containing a Strep-II affinity tag was obtained for the reduced, diamagnetic, form of the protein. This information will be essential to understand the role of MtoD in the EET processes in *S. lithotrophicus* ES-1.

## Methods and experiments

### Construction of bacterial strains

Strains, primers and plasmids used in this study are listed in Table 1. The gene *mtoD* was kindly provided by Thomas A. Clarke from the University of East Anglia, Norwich, UK. The sequence of the signal peptide derived from the periplasmic monoheme cytochrome MtoD from *S. lithotrophicus* ES-1 was replaced by the sequence of the signal peptide of OmpA from *E. coli*. The DNA fragment coding for OmpA was amplified from the genomic DNA of *E. coli* (DH5α) using primers 1 and 2, while the MtoD protein with the Strep-II tag was amplified using primers 3 and 4 (Table 1). The OmpA and MtoD fragments were purified and cloned in the NcoI restriction site of the pBAD202/D-TOPO vector (Invitrogen, Carlsbad, CA, USA) using the NEBuilder Assembly kit (New England BioLabs). The resulting plasmid (pBAD202::*mtoD*) was transformed into chemically competent *E. coli* strain JM109(DE3) co-transformed with vector pEC86, which contains the *ccmABCDEFGH* genes (Thöny-Meyer et al. 1995).

**Table 1:** Bacterial strains, plasmids, and primers used in this study

Strains, plasmids, primers	Relevant characteristics	Source
*Strains*		
*E. coli* DH5α	Host for cloning	IBN Lab Strains Collection
*E. coli* JM109(DE3)	*E. coli* strain co-transformed with vector pEC86, which contains the *ccmABCDEFGH* genes	IBN Lab Strains Collection
*Plasmids*		
pBAD202/D-TOPO	Expression vector, Km^r^	Invitrogen
pBAD202::*mtoD*	pBAD202/D-TOPO containing the OmpA signal peptide from *E. coli*, the *mtoD* gene and a Strep-II tag sequence.	This study
***Primers***	***Sequence (5’ to 3’)***	
Primer 1	GAGATATACATACCATGAAAAAGACAGCTATCGCGATTGC	This study
Primer 2	CAGCGTCGACATCGACAGCTGCCTGCGCAACGGTAGC	This study
Primer 3	GCTACCGTTGCGCAGGCAGCTGTCGATGTCGACGCTG	This study
Primer 4	TATCAGATCCCATGCTACTTCTCGAATTGTGGATGAGAC	This study

### Protein expression and purification

MtoD (*mtoD* gene containing a Strep-II tag − residues 28–117 and residues 118−125, respectively) was produced as unlabeled, single ^15^N-labeled, uniform double ^15^N,^13^C-labeled and selective-unlabeled ^15^N-labeled samples. The *E. coli* strains JM109(DE3) harboring pEC86 and pBAD202::*mtoD* were grown aerobically at 37 ºC in Lysogeny Broth (LB) medium containing 50 µg/cm^3^ kanamycin and 34 µg/cm^3^ chloramphenicol in 5.0 dm^3^ Erlenmeyer flasks containing 2.0 dm^3^ of medium at 150 rev./min to an optical density (OD_600nm_) of 1.0. This culture was then processed as follow: (a) to produce unlabeled MtoD: 10 mM L-arabinose was added and the cells were allowed to grow under the same conditions for 8 h before harvesting; (b) to produce ^15^N-MtoD: the cells were collected by centrifugation, washed twice with 250 cm^3^ of a salt solution containing 12.8 g/dm^3^ Na_2_HPO_4_, 3.0 g/dm^3^ KH_2_PO_4_ and 0.50 g/dm^3^ NaCl, resuspended in minimal media (in a ratio of 250 cm^3^ of minimal medium for each dm^3^ of LB medium) supplied with 1.0 g/dm^3^ of ammonium sulfate (^15^N_2_, 99%) as nitrogen source, (together with 2 mM MgSO_4_, 0.1 mM CaCl_2_, 0.05 mM FeSO_4_ and 0.2 g/L yeast extract), grown for 8 h at 37 ºC in the presence of 10 mM L-arabinose and harvested by centrifugation; (c) to produce uniform ^15^N,^13^C-MtoD: as described in (b) but also supplied with 2.0 g/dm^3^ [U–^13^C_6_] D-glucose as carbon source; (d) to produce selective-unlabeled ^15^N-MtoD: as described in (b) but each growth also supplied with one of four different amino acids in an unlabeled form (Asn, Cys, His or Lys, at 1.0 g/ dm^3^).

All bacterial cells were harvested by centrifugation at 10,000 *g* for 15 min at 4 ºC. The cell pellet was resuspended in 100 mM Tris-HCl (pH 7.4) containing EDTA-free protease inhibitor cocktail (Roche) and DNase I (Sigma). The disruption of the cells was achieved by three passages through a French Press at a pressure of 1,000 psi (6.89 MPa). The crude extract was centrifuged at 200,000 g for 1 h at 4 ºC (Beckman Coulter Optima LE-80 K). The supernatant containing the soluble protein fraction was loaded into a 5 mL Strep-Tactin® column (IBA) previously equilibrated with 100 mM Tris-HCl, pH 7.4, 150 mM NaCl and the protein was eluted using Elution Buffer (100 mM Tris-HCl, pH 7.4, 150 mM NaCl, 2.5 mM desthiobiotin). Eluted fractions were analyzed by SDS-PAGE (12% gel) stained for heme proteins (Francis and Becker, 1984) and BlueSafe. The purity index of the sample was defined by the A_Soret peak_/A_280nm_ ratio using UV-visible spectroscopy measurements. The final purified MtoD was concentrated at 4 ºC using an Amicon Ultra Centrifugal Filter (Millipore) with a 5 kDa cutoff. Protein concentration was estimated using the absorption coefficient, ε_409nm_, of 125,000 M^− 1^cm^− 1^ per heme for the oxidized state of the protein (Massey 1959).

### NMR experiments

Samples for NMR experiments with concentrations between 0.1 and 1.5 mM were prepared in ^2^H_2_O (99.9% atom) and 90% H_2_O / 10% ^2^H_2_O (99.9 atom %). To obtain the fully reduced state of the protein, small amounts of solid sodium dithionite were added to the sample, in an argon atmosphere. NMR experiments were performed at 298 K on a NEO 500 MHz NMR spectrometer (Bruker, Rheinstetten, Germany) equipped with a 5 mm TCI C/N prodigy cryoprobe, on a Bruker Avance 800 MHz and on a Bruker 950 MHz spectrometers equipped with a helium cold TCI cryoprobe, and on a Bruker 1,200 MHz spectrometer equipped with a TXO 5 mm cryoprobe. Assignment of the proton heme signals was performed using a 0.1 mM sample of unlabeled MtoD lyophilized twice using ^2^H_2_O (99.9 atom%) prepared in 20 mM potassium phosphate buffer at pH 7.6 with 80 mM NaCl. NMR spectra obtained before and after the lyophilization were identical, showing that the protein integrity was not affected by the procedure. 2D-nuclear Overhauser effect spectroscopy (NOESY) (50, 200 and 400 ms, mixing time) spectra and 2D-total correlation spectroscopy (TOCSY) (60 ms mixing time) spectra were collected. For backbone and side chain assignments, single ^15^N-labeled, uniform double ^15^N,^13^C-labeled, and selective-unlabeled ^15^N-labeled samples with concentrations between 0.1 and 1.5 mM were prepared in 20 mM potassium phosphate buffer at pH 6.5 with 50 mM NaCl. A set of double and triple resonance experiments was performed: (i) ^13^C- and ^15^N-HSQC and HSQC-NOESY, HNCA, HNCO, HNCACO, CBCA(CO)NH, CBCANH, HNHA, HCCH-TOCSY (Cavanagh et al. 2007) for ^15^N,^13^C-MtoD sample and (ii) ^15^N-HSQC for the ^15^N-MtoD and selective-unlabeled ^15^N-MtoD (Table S1). ^1^H chemical shifts were calibrated using the water signal as an internal reference and both ^13^C and ^15^N chemical shifts were calibrated through indirect referencing (Wishart et al. 1995). All NMR spectra were processed using Topspin 4.1.1 software (Bruker Biospin, Karlsruhe, Germany). Sparky (TD Goddard and DG Kneller, Sparky 3, University of California, San Francisco, USA) was used for heme assignment and CARA (Keller 2004) was used for the backbone and side chain assignments.

## Results

### Extent of assignments and data deposition

#### Production of MtoD

The production of *c*-type cytochromes requires specific cell machinery, such as the cytochrome *c* maturation system and Sec translocation system (Verissimo and Daldal 2014). To ensure that the target protein MtoD is recognized by the *E. coli* Sec translocation system, the signal peptide of OmpA was used. It is known that the outer-membrane protein OmpA from *E. coli* is constitutively expressed in high levels in *E. coli* under aerobic conditions, containing the ideal signal peptide for the heterologous expression of proteins, including MtoD (Low et al. 2013). To facilitate the purification process, the protein was engineered to carry a C-terminal Strep-II affinity tag. SDS-PAGE analysis confirmed the purity of MtoD, running as a single band with an apparent molecular weight of 11 kDa (Figure S1A) and staining positively for the covalent attachment of the heme (Figure S1B). Pure MtoD presented an absorbance ratio of A_Soret Peak_/A_280nm_ of 5 (Figure S1C).

### NMR assignments

#### Assignment of heme resonances

**Fig. 1:**
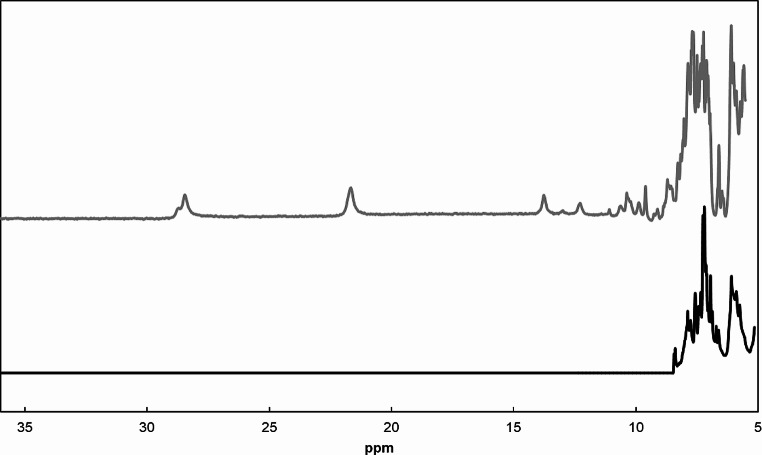
^**1**^H NMR spectra of cytochrome MtoD in the reduced and oxidized state downfield of the residual solvent water signal. Spectrum in the reduced state (black line) was obtained by the addition of small amounts of sodium dithionite to the oxidized (gray line) sample

To properly map the interacting docking regions within a redox complex established with MtoD, it is necessary to assign the protein’s backbone NMR signals and the heme substituent NMR signals. The crystal structure of MtoD previously determined (Beckwith et al. 2015), reveals that the heme has bis-histidinyl axial coordination. Given the differences between the spin state of the system (i.e. diamagnetic (Fe(II), S = 0) in the reduced state and paramagnetic (Fe(III), S = 1/2) in the oxidized state) (Querci et al. 2024; Trindade et al. 2022), 1D ^1^H NMR spectra of cytochrome MtoD display distinct features in the reduced and oxidized states (Fig. 1). In the reduced state, the signals are sharp and spread over the 10 to − 1 ppm region, while in the oxidized state the signals are broader and span a wider spectral region ranging from 29 to − 2 ppm. In fact, because the heme is diamagnetic in the reduced state, the signals of the heme substituents can be observed in typical regions: 9 − 10 ppm for meso protons (H5, H10, H15, and H20, according to the IUPAC-IUB nomenclature for tetrapyrroles (Figure S2); 5 − 7 ppm for thioether methines (H3^1^ and H8^1^); 2 − 4 ppm for methyl groups (M2^1^, M7^1^, M12^1^, and M18^1^); and 1 − 3 ppm for thioether methyls (M3^2^ and M8^2^) (Silva and Louro 2017).

The heme proton signals were assigned using the 2D ^1^H NMR spectra (NOESY and TOCSY) acquired for the non-labeled sample prepared in ^2^H_2_O, as previously described (Silva and Louro 2017) (Figure S3). The carbon signals were assigned using the 2D ^1^H,^13^C-HSQC and 3D ^13^C-edited [^1^H,^1^H]-NOESY spectra. The assignment of the heme signals is shown in Table 2.

**Table 2:** Assignment of proton and carbon NMR resonances of the heme substituents of MtoD. The heme substituents are numbered according to the IUPAC-IUB nomenclature (Figure S2)

Substituent	Chemical shifts of heme protons (ppm)	Substituent	Chemical shifts of heme carbons (ppm)
H5	9.43	CAA	26.09
H10	8.88	CAB	34.84
H15	9.57	CAC	38.54
H20	9.17	CAD	25.21
M2^1^	3.25	CBB	24.32
M7^1^	3.64	CBC	22.31
M12^1^	2.52	CBD	44.87
M18^1^	3.53	CHA	99.76
H3^1^	5.82	CHB	99.12
H8^1^	6.14	CHC	100.75
M3^2^	1.87	CHD	100.73
M8^2^	2.2	CMA	14.86
P17^1^_1_	4.26	CMB	15.02
P17^1^_2_	3.51	CMC	15.02
P13^1^_1_	4.34	CMD	11.18
P13^1^_2_	3.82		
P13^2^_1_	3.27		
P13^2^_2_	2.92		

### Assignment of protein resonances

**Fig. 2:**
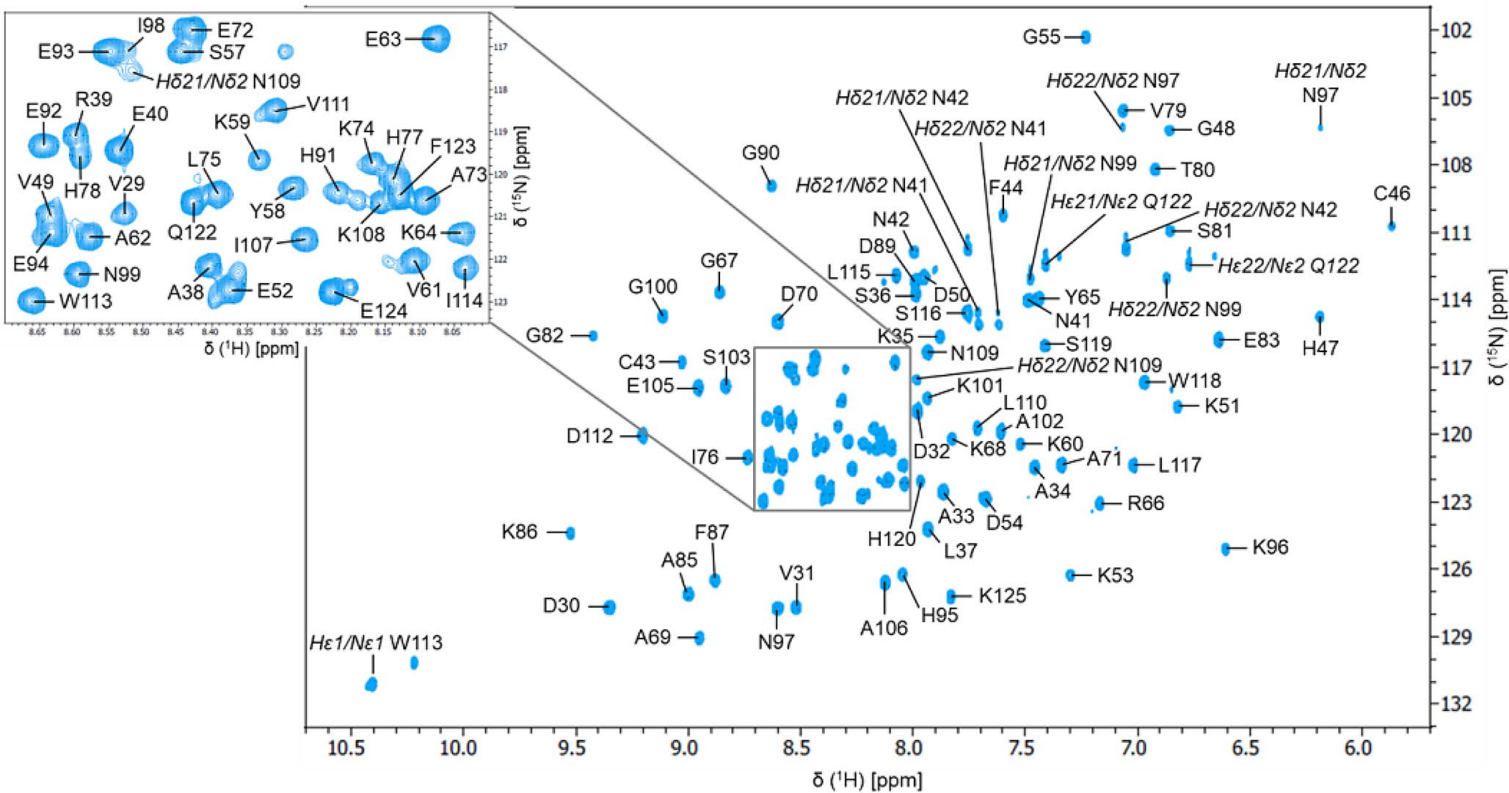
^**15**^**N**,^**1**^H-HSQC spectrum of MtoD. The spectrum was recorded at a proton resonance frequency of 800 MHz at 298 K, in 20 mM potassium phosphate buffer (90% H_2_O / 10% ^2^H_2_O) at pH 6.5 with 50 mM NaCl concentration. Resonance assignments are labelled in black according to the native sequence of MtoD. The insert expands the more crowded region of the spectrum

The combined analysis of 2D ^1^H,^15^N-HSQC and the series of 3D NMR spectra (3D CBCANH, 3D CBCA(CO)NH, 3D HNCO, 3D HNCA, 3D HNCACO) led to the near-complete assignment of backbone ^15^N (94%), ^1^H_N_ (98%), ^13^C_α_ (100%), ^13^C_β_ (100%) and ^13^CO (97%). The only residues unobserved in the ^15^N HSQC experiment were Lys 45 and Lys 84. Figure 2 shows the 2D ^1^H,^15^N-HSQC NMR spectrum of labeled MtoD with the HN assignments of backbone and side chains of asparagine (N41, N42, N97, N99, N109) and glutamine (Q122) residues.

The 2D ^1^H,^13^C-HSQC spectrum, combined with 3D HCC(H) TOCSY spectrum, allowed the assignment of the aliphatic protons and remaining aliphatic carbon signals of the protein (^1^H_α_, ^1^H_β_, ^1^H_γ_, ^1^H_δ_, ^1^H_ε_ and ^13^C_γ_, ^13^C_δ_, ^13^C_ε_). The total extent of the assignment for the ^1^H, ^13^C and ^15^N, is 83%, 83% and 82%, respectively. The ^1^H, ^13^C and ^15^N chemical shifts have been deposited in the BioMagResBank (http://www.bmrb.wisc.edu) under BMRB accession number 51671.

### Analysis of chemical shifts

**Fig. 3:**
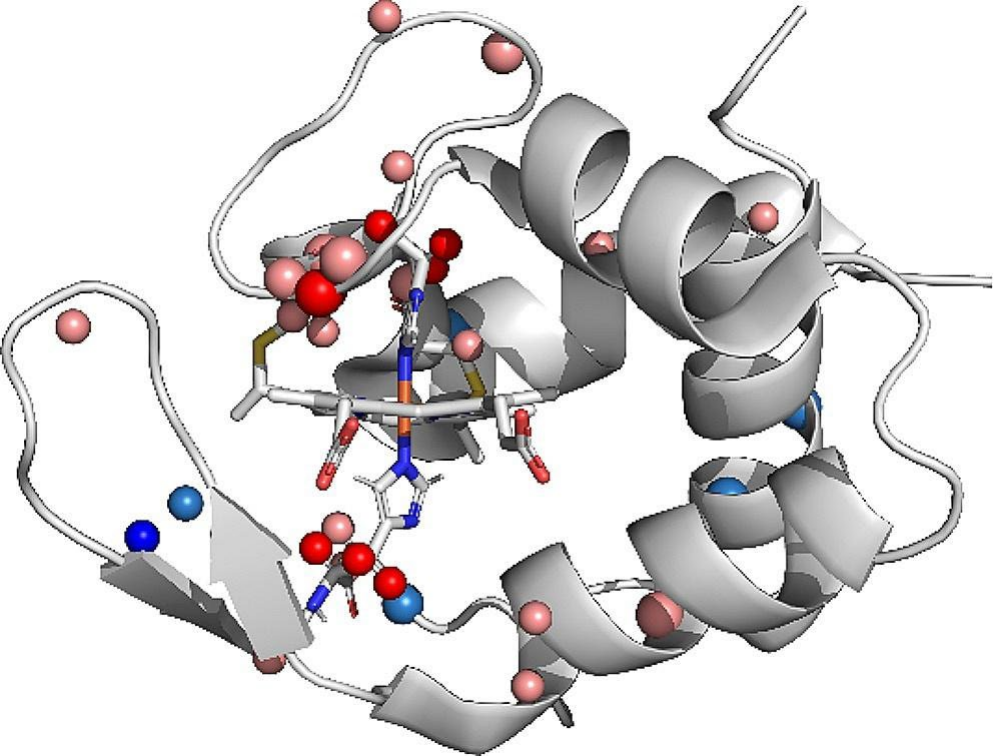
Chemical shifts deviating from the statistics reported in the BMRB database shown in the crystallographic protein structure. Atoms deviating from the average shift by more than two standard deviation values are, respectively, shown in light blue (positive shift) and pink (negative shift); atoms deviating by more than three standard deviation values are, respectively, shown in blue (positive shift) and red (negative shift). A larger sphere radius was used for heteroatoms

The ring-current effects generated by the heme group are significantly stronger than those produced by the amino acid aromatic side chains. Consequently, the nuclei located close to the heme group are subject to a strong ring-current contribution on their observed shifts and may significantly deviate from the expected values. The most affected nuclei are those belonging to the axial histidine. In fact, the His H_β2_ and H_β1_, and H_α_ signals are strongly up-field shifted appearing in the − 0.12–1.39 ppm and 2.83–3.20 ppm range, respectively, with sizable deviations from the values that are typically found in the absence of heme-ring current shifts (around 2.7–3.5 ppm for H_β_ and around 4.2–5.0 ppm for H_α_). The chemical shifts of other nuclei located in the neighborhood of the heme group are also significantly affected as is the case of Cys 43 H_β_, Cys 46 H_N_ and H_β_, Gly 48 H_N_, Lys 96 H_N_, H_α_, H_β_, and H_ε_, which experience a shielding effect with respect to the random coil values. Since the monoheme cytochrome MtoD is relatively small (11 kDa), most nuclei are located in the vicinity of the heme group, and therefore this effect is extended to the proton, nitrogen, and carbon atoms of other amino acids. Indeed, Fig. 3 shows, for overall backbone atoms and for the side chains of heme bound residues, atoms with shifts that deviate from the expectation values (https://bmrb.io/ref_info/csstats.php?restype=aa&amp;set=filt) .

The observed chemical shifts of nuclei located in the vicinity of the heme group are affected differently, depending on their distance from the heme and position relative to the heme plane (Cross and Wright 1985; Johnson and Bovey 1958). Consistent with the structure of the heme pocket, a predominance of shielding contributions are observed along the bis-histidine axis of the system.

**Fig. 4:**
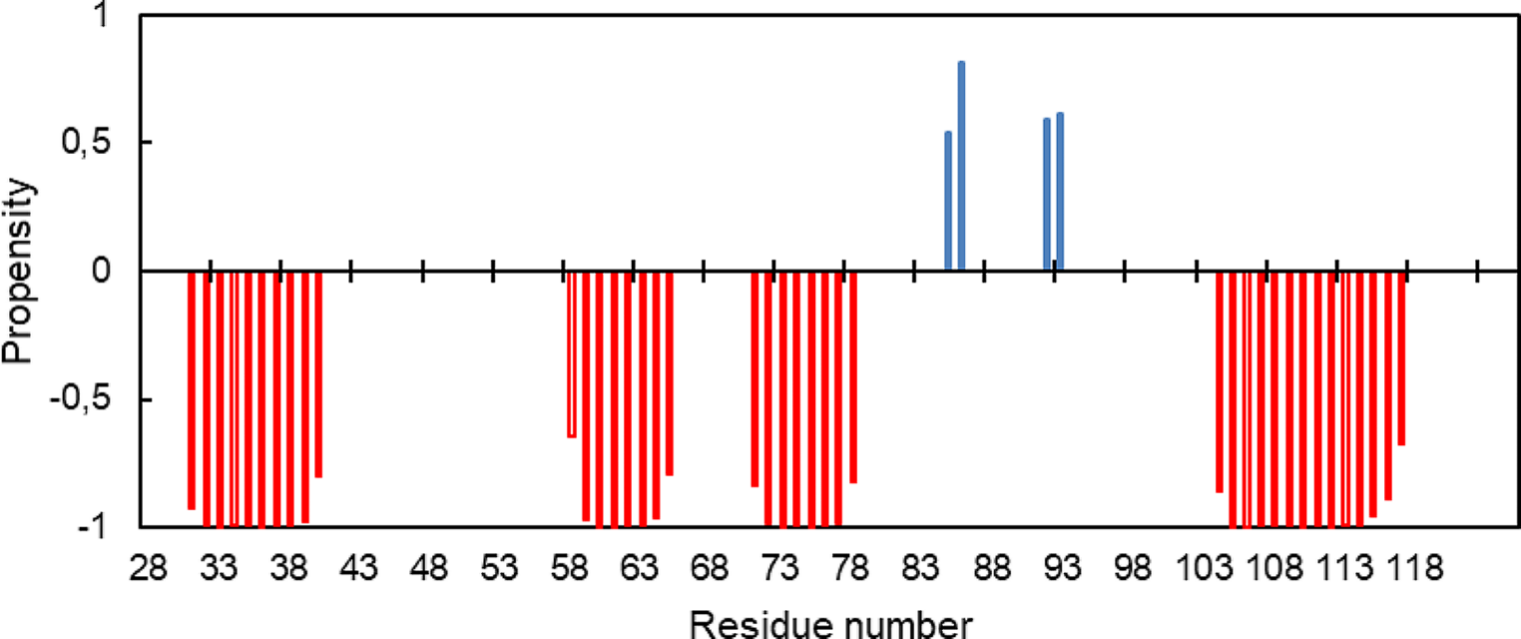
Secondary structure elements of MtoD predicted by TALOS + software. α-helices are in red, β-strands in blue and the loop segments are not shown

Despite the caveat of the effect of the heme ring current shifts, the spectral position of the assigned backbone signals was then used to predict the protein secondary structure elements in solution using the TALOS + software (Shen et al. 2009) (Fig. 4). The data indicate that the dominant secondary structural elements in this protein are α-helices. The prediction closely matches that of the crystal structure of the protein (Beckwith et al. 2015).

## Conclusion

The complete assignment of the heme proton and carbon (quaternary) signals together with a near-complete assignment of ^1^H, ^13^C and ^15^N backbone and side chain resonances obtained in the present work constitute important foundations to investigate and structurally map the interactions between MtoD and physiological partners. NMR-based biomolecular interaction studies between MtoD and its redox partners will contribute to characterize the molecular choreography that underpins the EET pathway of *S. lithotrophicus* ES-1, a knowledge that is still lacking in the quest to understand the biogeochemical cycling of metals in the environment.

## Electronic supplementary material

Below is the link to the electronic supplementary material.

Supplementary Material 1

## Data Availability

Assignment deposited in BMRB, ID 51671.
