# Supplementary material for: Resonance assignments of cytochrome MtoD from the extracellular electron uptake pathway of sideroxydans lithotrophicus ES-1
Source: Biomol NMR Assign. 2024 Jun 7;18(2):139–46. doi: 10.1007/s12104-024-10180-8 (PMC11511738; doi:10.1007/s12104-024-10180-8)
Supplement: Supplementary file 1 — Supplementary Material 1 [file 12104_2024_10180_MOESM1_ESM.docx]

**
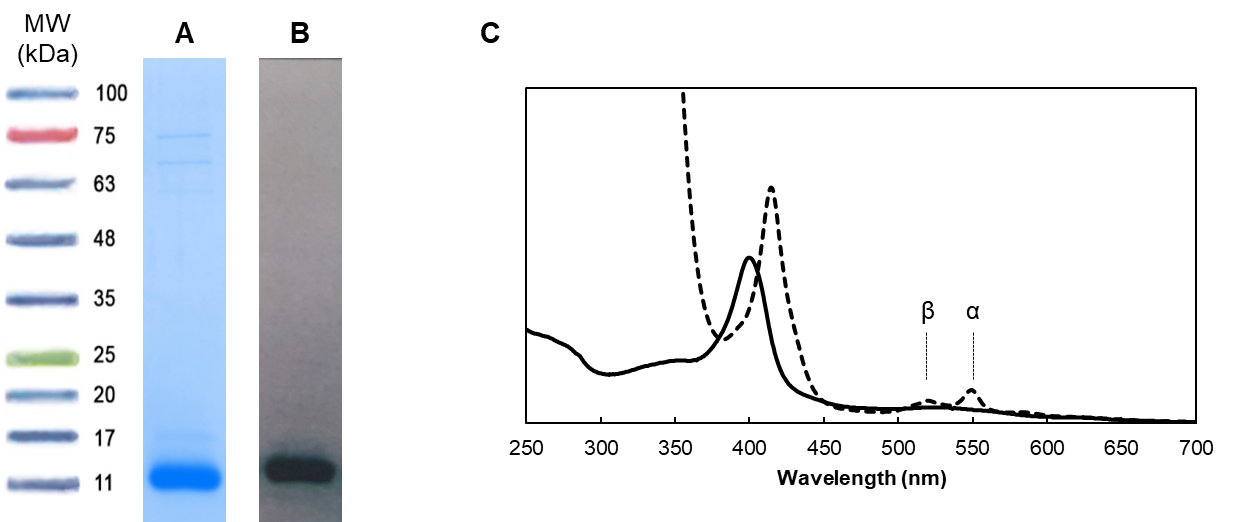
**

**Figure S1. Isolation of MtoD.** **(A)** Blue-safe and **(B)** heme-stained SDS-PAGE of purified MtoD. **(C)** UV-Visible absorption spectra in the oxidized (solid line) and reduced (dashed line) state. Spectrum in the reduced state was obtained by the addition of small volumes of concentrated sodium dithionite solution to the oxidized sample.

**Figure S2. Schematic representation of a *c*-type heme.** The heme proton substituents, represented in black, are numbered according to the IUPAC-IUB nomenclature (Moss et al., 1995, Silva and Louro, 2017), with M, H, and P designating methyl (CH_3_), hydrogen and propionate (CH_2_) groups, respectively. The heme carbon substituents, represented in gray, are designated by the PDB HEC nomenclature.

**Figure S3. Heme assignment strategy applied for cytochrome MtoD in the reduced state.** The spectra were recorded for the non-labeled sample at a proton resonance frequency of 500 MHz at 298 K, in 20 mM potassium phosphate buffer (^2^H_2_O) at pH 7.65 with 80 mM NaCl concentration. (A) 50 ms NOESY spectrum. (B) 200 ms NOESY spectrum. (C) 400 ms NOESY spectrum. The heme connectivities are highlighted in the spectra numbered according to the IUPAC-IUB nomenclature (Moss et al., 1995).

**Table S1. List of experiments collected to perform the sequence specific assignment.** Main parameters and magnetic fields used are reported.

| **Experiments** | **Time domain data size** | | | **Spectral width** | | | **ns** | **Delay time** | **Magnetic field** |
| --- | --- | --- | --- | --- | --- | --- | --- | --- | --- |
|  | **(points)** | | | **(ppm)** | | |  | **(s)** | **(MHz)**  **(^1^H Larm freq)** |
|  | **t_1_** | **t_2_** | **t_3_** | **F_1_** | **F_2_** | **F_3_** |  |  |  |
| **[^1^H,^1^H]-NOESY** | 512 | 2048 |  | 15.6  (^1^H) | 15.6  (^1^H) |  | 64 | 0.5 | 500 |
| **[^1^H,^1^H]-TOCSY** | 512 | 2048 |  | 15.6  (^1^H) | 15.6  (^1^H) |  | 64 | 0.5 | 500 |
| **^1^H,^15^N-HSQC** | 128 | 2048 |  | 40.0  (^15^N) | 13.0  (^1^H) |  | 8 | 0.1 | 800 |
| **^1^H,^13^C-HSQC** | 256 | 2048 |  | 80.0  (^13^C) | 16.0 (^1^H) |  | 64 | 1.0 | 1200 |
| **HNCA** | 128 | 64 | 698 | 30.0  (^13^C) | 35.0  (^15^N) | 16.0  (^1^H) | 16 | 0.2 | 800 |
| **HNCO** | 128 | 64 | 698 | 14.0  (^13^C) | 35.0  (^15^N) | 16.0  (^1^H) | 16 | 0.2 | 800 |
| **HNCACO** | 128 | 40 | 2048 | 14.0  (^13^C) | 35.0  (^15^N) | 16.0  (^1^H) | 24 | 1.0 | 800 |
| **CBCACONH** | 128 | 40 | 2048 | 80.0  (^13^C) | 35.0  (^15^N) | 16.0  (^1^H) | 40 | 1.0 | 800 |
| **CBCANH** | 128 | 40 | 2048 | 75.0  (^13^C) | 35.0  (^15^N) | 16.0  (^1^H) | 48 | 1.0 | 800 |
| **HNHA** | 40 | 128 | 2048 | 35.0  (^15^N) | 13.9  (^1^H) | 13.9  (^1^H) | 16 | 1.0 | 800 |
| **HBHACONH** | 128 | 40 | 2048 | 13.9  (^1^H) | 40.0  (^15^N) | 13.9  (^1^H) | 20 | 1.0 | 800 |
| **(H)CCH-TOCSY** | 124 | 64 | 2048 | 80.0  (^13^C) | 80.0  (^13^C) | 13.9  (^1^H) | 16 | 1.0 | 800 |
| **^15^N-edited [^1^H,^1^H]-NOESY** | 220 | 48 | 2048 | 14.0 (^1^H) | 40.0  (^15^N) | 14.0 (^1^H) | 16 | 1.0 | 950 |
| **^13^C-edited [^1^H,^1^H]-NOESY** | 220 | 56 | 2048 | 14.0 (^1^H) | 80.0  (^13^C) | 14.0 (^1^H) | 16 | 1.0 | 950 |
